# Endoplasmic Reticulum Stress and Unfolded Protein Response Pathways: Potential for Treating Age-related Retinal Degeneration

**Published:** 2012-01

**Authors:** Mohammad Haeri, Barry E Knox

**Affiliations:** SUNY Eye Institute, Departments of Neuroscience and Physiology and Ophthalmology, SUNY Upstate Medical University, Syracuse, NY, USA

**Keywords:** ER Stress, Unfolded Protein Response, Age-related Retinal Degeneration, Misfolded Protein, Protein Aggregation, Retinal Degenerative Diseases

## Abstract

Accumulation of misfolded proteins in the endoplasmic reticulum (ER) and their aggregation impair normal cellular function and can be toxic, leading to cell death. Prolonged expression of misfolded proteins triggers ER stress, which initiates a cascade of reactions called the unfolded protein response (UPR). Protein misfolding is the basis for a variety of disorders known as ER storage or conformational diseases. There are an increasing number of eye disorders associated with misfolded proteins and pathologic ER responses, including retinitis pigmentosa (RP). Herein we review the basic cellular and molecular biology of UPR with focus on pathways that could be potential targets for treating retinal degenerative diseases.

## ENDOPLASMIC RETICULUM STRESS AND PROTEIN FOLDING

The endoplasmic reticulum (ER) is the principal site for synthesis and maturation of secretory and transmembrane proteins. More than 30% of cellular proteins are synthesized in the ER.^1^ Folding is the most critical step in protein synthesis. Any delay in or impairment of protein folding and processing results in accumulation of misfolded protein triggering ER stress which is a response characterized by ER distension and perturbation in ER homeostasis.^2^ Accumulation of misfolded proteins and/or their aggregation are toxic to the cell and impair normal cellular function. To cope with any perturbation by unfolded or misfolded proteins, the ER induces a cascade of reactions called the unfolded protein response (UPR).^3^ ER stress is the precursor of UPR, however these terms have been used interchangeably and will be used as such throughout this review.

Protein misfolding is the basis for a variety of disorders known as ER storage or conformational diseases.^4^,^5^ However, recent studies show that UPR is activated during normal cellular events such as B-cell differentiation,^6^ muscle differentiation,^7^ or viral infections.^8^,^9^ The common theme in these events which trigger the UPR response is the build-up of unfolded proteins.^10^–^15^

**Figure 1.**
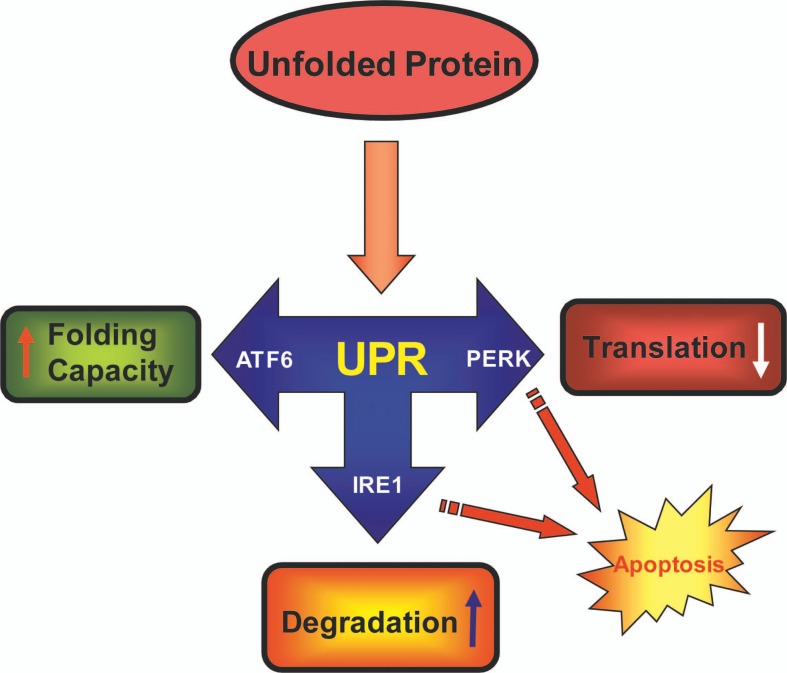
Major unfolded protein response (UPR) pathways. The influx of unfolded proteins in the endoplasmic reticulum (ER) induces UPR. UPR is relayed to the cell by activation of three receptors: IRE1, PERK and ATF6. Induction of these pathways starts processes to (1) decrease the load of protein by translational attenuation, (2) to increase the folding capacity of the ER, and (3) to decrease the protein load in the ER by degradation of unfolded proteins. Cells which fail to restore their normal cellular function experience prolonged activation of UPR resulting in apoptosis.

Generally, cellular responses in UPR are composed of (1) translational attenuation to prevent further production of unfolded proteins,^16^ (2) transcriptional induction of ER resident proteins (chaperones and foldases) to further assist protein folding,^17^ (3) induction of ER-associated degradation (ERAD) machinery to handle misfolded proteins and reduce the burden on ER folding capacity,^18^ and (4) enlargement of the ER to deal with the large load of unfolded proteins.^19^ The successful execution of compensatory responses to stress by these mechanisms restores normal cell function and survival. Alternatively, irreversibly stressed cells will undergo apoptosis in response to prolonged UPR (Fig. 1).

**Figure 2.**
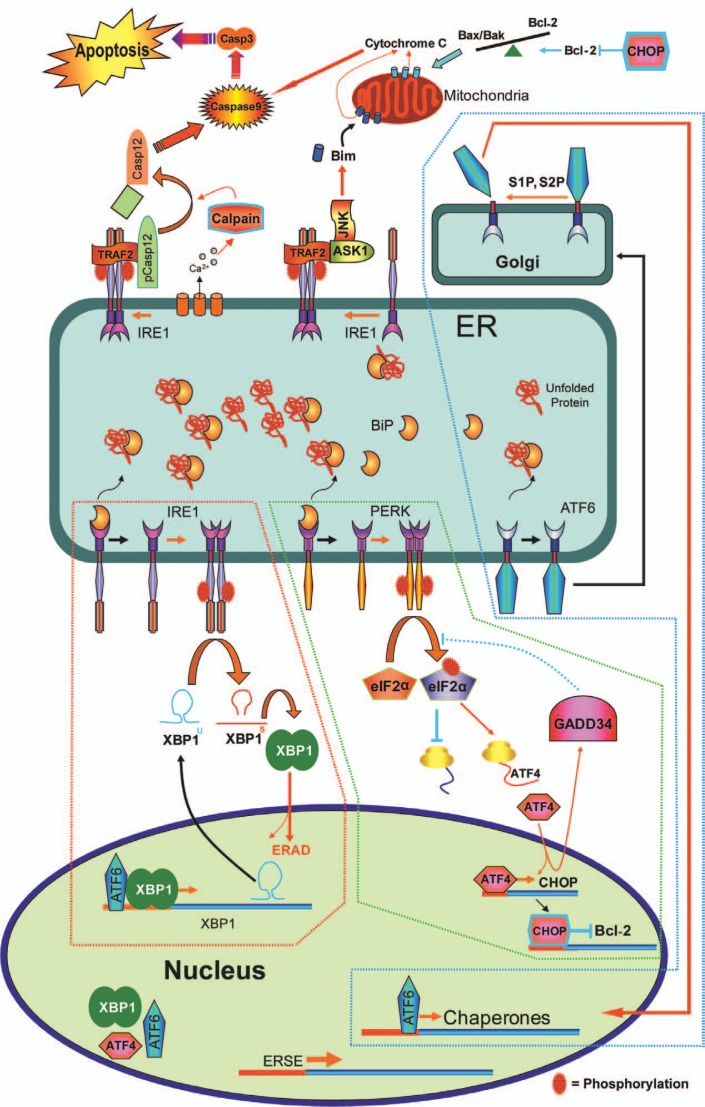
Unfolded protein response (UPR). The ER stress response is mediated by three receptors located in the ER membrane. The IRE1 pathway is activated by the release of BiP from IRE1 which is followed by dimerization and autophosphorylation of its cytoplasmic domain. The activated IRE1 receptor triggers its intrinsic RNase activity, which splices out a 26-nucleotide intron from XBP1 mRNA and creates a translational frameshift leading to production of an active transcription factor, XBP1. XBP1 activates the transcription of ERAD-related proteins (red-dotted area). Chronic activation of IRE1 leads to recruitment of TRAF2 followed by activation of ASK1, p38 and JNK. Activated JNK translocates to the mitochondrial membrane and promotes phosphorylation of Bim, which is associated with Bax-dependent release of cytochrome c and activation of the caspase cascade. The PERK pathway is activated by the release of BiP generating a dimerized-phosphorylated PERK enzyme which reduces the activity of the eIF2α by phosphorylation of its α-subunit. Although the phosphorylation of eIF2α generally attenuates protein synthesis/translation, it promotes the translation of ATF4 upon ER stress. ATF4 activates the transcription of chaperones, ERAD machinery and ER-stress-induced pro-apoptotic factors, such as CHOP and GADD34 and ATF3 (green-dotted area). In the ATF6 pathway, the release of BiP from the ATF6 receptor frees the receptor to be transported to the Golgi. Once in the Golgi, the cytosolic domain is cleaved by S1P and S2P, releasing a bZIP transcription factor, ATF6. ATF6 translocates to the nucleus where it activates the transcription of BiP, XBP1, CHOP and P58IPK (blue-dotted area).

The ER stress response is mediated by three receptors located in the ER membrane. Translational attenuation in the UPR is mediated by the double-stranded RNA-activated protein kinase (PKR)-like endoplasmic reticulum kinase (PERK) signalling pathway. PERK receptors sense the presence of unfolded proteins^20^ and reduce the activity of the ribosomal initiating factor (eIF2α) by phosphorylation of its α-subunit.^16^ Transcriptional activation of ER-resident proteins is mediated by activating transcription factor 6 (ATF6)^21^ and inositol requiring kinase 1 (IRE1) receptors. In a similar mechanism to PERK receptors, ATF6 and IRE1 receptors sense the accumulation of misfolded proteins and induce the transcription of ER-resident proteins to assist folding and to degrade misfolded proteins. ATF6 activates the transcription of molecular chaperones such as BiP, GRP94^17^ and PDI^22^. IRE1 induces the synthesis of a potent X-box-binding protein 1 (XBP1) and consequently activates the transcription of ER associated degradation (ERAD) proteins such as EDEM.^23^,^24^ IRE1 also activates the transcription of other UPR target genes, such as BiP, through XBP1 activation^25^ (Fig. 2).

To assist protein folding in the UPR, BiP is the first target for upregulation. BiP is a molecular chaperone with high affinity for short hydrophobic peptides on the surface of unfolded proteins. The binding and releasing of these peptides from BiP assist the folding of the substrate protein.^26^,^27^ BiP is also a sensor for unfolded proteins in the ER. An overload of unfolded proteins in the ER titrates BiP away from the luminal domains of IRE1, PERK and ATF6 receptors, which results in their further homodimerization, autophosphorylation and activation^16^,^20^,^28^. The dissociation of BiP from the ATF6 luminal domain leaves ATF6 free to be transported to the Golgi where it is cleaved to become an active transcription factor^29^ (Fig. 2). Downstream UPR target genes are regulated by two distinct consensus response elements, the *cis*-acting ER stress response element (ERSE) and the unfolded protein response element (UPRE) both of which are located in the promoter of genes involved in UPR. Binding of ATF6^21^ and XBP1 to these elements promotes transcription of downstream UPR genes^30^,^31^.

The IRE1, PERK and ATF6 receptors initiate pathways, which comprise the three arms of the UPR. Located in the ER membrane, these receptors have a transmembrane domain spanning the ER membrane, a cytosolic or nucleoplasmic domain, and a luminal domain that has a binding site for BiP. IRE1 receptor is a type I transmembrane protein kinase receptor which has site-specific endoribonuclease (RNase) activity in its cytosolic domain.^32^–^35^ IRE1 is highly conserved in eukaryotes.^28^,^32^,^33^,^36^–^38^ Two types of IRE1 receptors have been found in mammals. IRE1α is ubiquitously expressed in all tissues, but the expression of the second type, IRE1β, is limited to the gut.^20^,^34^,^35^,^39^ Deletion of IRE1α is lethal in mice; however, IRE1β^−/−^ mice are viable.^20^,^39^ The luminal domain of the IRE1 receptor binds to BiP. Accumulation of unfolded proteins in the ER, results in BiP release from the IRE1 luminal domain which in return causes dimerization and autophosphorylation of IRE1. Phosphorylation of the cytosolic domain of the IRE1 receptor activates its RNase activity.^30^,^31^,^40^–^42^ In mammals, XBP1 mRNA is the substrate for IRE1 endoribonuclease.^30^,^31^,^40^ XBP1, a bZIP (basic region and leucine zipper)-domain-containing transcription factor of the ATF/CREB (cyclic-AMP-responsive-element-binding protein) family, binds to ERSE.^21^,^43^ During ER stress, IRE1 endoribonuclease splices out a 26-nucleotide intron from XBP1 mRNA and creates a translational frameshift. This spliced form of XBP1 becomes an active transcription factor.^15^,^30^,^31^,^40^ XBP1 then translocates to the nucleus and induces the transcription of genes involved in protein folding and degradation 24,40 44–48 (Fig. 2).

The PERK receptor is a type I transmembrane protein kinase receptor which is activated by ER stress with the release of BiP from its luminal domain. Release of BiP from PERK, results in its oligomerization with subsequent autophosphorylation of its cytosolic domain.^16^,^20^,^21^,^49^ Activated PERK receptors phosphorylate the α-subunit (S51) of eIF2 (eIF2α), which inhibits 80S ribosome assembly and consequently attenuates protein synthesis/translation.^16^,^50^,^51^ The inhibition of ribosomal assembly reduces the influx of newly synthesized proteins to an already saturated ER. In contrast, phosphorylation of eIF2α promotes the translation of ATF4 upon ER stress.^11^,^52^,^53^ ATF4 is a transcription factor which activates the transcription of chaperones, ERAD machinery and other genes involved in the oxidative stress response and ER stress-induced apoptosis^20^,^54^–^56^, such as C/EBP homologous transcription factor (CHOP), growth arrest and DNA damage gene 34 (GADD34)^20^,^57^–^59^ and ATF3^58^.

The ATF6 receptor is a type II transmembrane protein with a cytosolic/nucleoplasmic and an ER luminal domain.^60^ Mammals have two ATF6 homologs, ATF6α and ATF6β.^61^ There are two distinct ER stress-regulated Golgi localization sequences (GLS1 and GLS2) in the luminal domain of ATF6α and one GLS2 in that of ATF6β. BiP binds to GLS1 and retains the ATF6 receptor within the ER. Release of BiP from GLS1 upon ER stress results in ATF6 translocation to the Golgi.^62^ The cytosolic domain of ATF6 in the Golgi is cleaved by site-1 proteases (S1P and S2P) releasing a bZIP transcription factor, ATF6.^18^,^29^,^63^–^65^ ATF6 translocates to the nucleus where it interacts with nuclear factor-Y and XBP1, binds to ERSE and UPRE elements and activates the transcription of *BiP*, *XBP1*, *CHOP* and *P58^IPK^* 18,21,66–69 (Fig. 2). Activated ATF6 also induces the expression of genes involved in protein folding and degradation.^63^, ^64^

## PRO-APOPTOTIC PATHWAYS IN ENDOPLASMIC RETICULUM STRESS

Prolonged ER stress seems to activate IRE1α, ATF6 and PERK pathways sequentially and deactivates them in the same order.^70^ If the UPR response fails to restore cellular homeostasis, the cell initiates apoptosis.^53^,^71^,^72^ Prolonged ER stress-induced apoptosis is an important pathologic element of neurodegenerative diseases, diabetes, renal diseases and atherosclerosis.^73^ Generally, apoptosis is known as the nuclear form of programmed cell death (PCD). Other types of PCDs are autophagic (type II)^74^–^76^ and cytoplasmic (type III) PCD^75^–^78^. The PCD associated with UPR is probably the nuclear form. Four known pro-apoptotic pathways have been implicated in ER-stress mediated apoptosis; 1) IRE1α-mediated activation of ASK1 (apoptosis signal-regulating kinase 1), 2) caspase-12^79^, 3) PERK/eIF2α induction of CHOP, and 4) BAK/BAX-induced Ca^2+^ release from the ER 35,80.

Apoptosis signalling is mediated by two major pathways. The extrinsic pathway is triggered by self-association of membrane receptors and recruitment of caspase-9 which results in initiation of a caspase cascade and finally apoptosis. The intrinsic pathway is triggered by dominance of pro-apoptotic over anti-apoptotic members of the Bcl-2 family. Anti-apoptotic members of the Bcl-2 family are Bcl-2 and Bcl-X_L_, and pro-apoptotic members are BH3-only classes including Bid and Bim as well as multidomain groups, such as Bax and Bak. A variety of apoptotic stimuli promote translocation of Bax and Bak from the cytosol into the mitochondria resulting in release of cytochrome c and activation of caspases.^81^

The IRE1/TRAF2/ASK1/JNK pathway is considered the dominant pro-apoptotic pathway in ER stress. It has been shown that IRE1α is able to recruit TNF receptor-associated factor 2 (TRAF2) to its cytosolic domain. TRAF2 can subsequently activate apoptosis signal-regulating kinase (ASK) 1, p38 and finally c-Jun amino-terminal kinase (JNK).^20^,^28^,^82^ Co-expression of ASK1 and JNK results in phosphorylation and inactivation of Bcl-2.^83^ Activated JNK has been shown to translocate to the mitochondrial membrane^84^ and promote phosphorylation of Bim, which is associated with Bax-dependent release of cytochrome c^85^,^86^. ASK1 can also be activated by reactive oxygen species (ROS) initiating apoptosis. ASK1 is inhibited by thioredoxin (TDX), a protein which inactivates ROS, until accumulation of ROS causes oxidation of TDX, thereby disinhibition of ASK1.^28^,^87^ IRE1α can also induce apoptosis through 1) interaction of the TNF receptor 1 (TNFR1) with TRAF2/ASK1 and subsequent activation of JNK and through 2) interaction of TRAF2 and the inhibitor of κB-kinase (IKK) which leads to activation of nuclear factor κB^88^,^89^.

The PERK/ATF4/CHOP pathway is the second pro-apoptotic mechanism triggered by prolonged ER stress^35^,^90^,^91^. CHOP is minimally expressed under non-stress conditions; however, its expression is moderately increased upon ER stress. CHOP expression has been shown to induce cell cycle arrest and/or apoptosis.^35^,^80^,^90^–^94^ Transcriptional activation of CHOP is mediated by the IRE1, PERK or ATF6^18^,^20^,^35^,^80^,^95^ pathways. CHOP is also activated post-translationally via p38 MAP kinase phosphorylation.^90^ It has been shown that CHOP expression sensitizes the cell to apoptosis by downregulation of BCL-2 and/or depletion of glutathione leading to accumulation of ROS in the cell^93^ (Fig. 2). CHOP expression promotes pro-apoptotic factors such as DR5, TRB3, BIM and GADD34 96–99. Association of CHOP with ER stress-induced apoptosis has been reported in diabetes,^80^,^99^ renal dysfunction,^35^ atherosclerosis,^100^ cardiac overload,^101^ colitis,^102^ and Parkinson’s disease^103^. CHOP is a major pro-apoptotic factor in ER stress and its deletion promotes cell survival. Several therapeutic approaches have targeted the PERK-CHOP pathway in a mouse model of ER stress to inhibit apoptosis. Blocking the phosphorylation of eIF2α by Salubrinal promoted cell survival in these mouse models implicated with ER stress.^104^

The PERK pathway is also induced by accumulation of ROS^105^,^106^. ROS are small molecules or free radicals (atoms, molecules or ions with unpaired electrons or open shelf configuration) formed by molecular oxygen.^107^ ROS have been linked to mediators of inflammation.^108^ Protein folding is an energy consuming process through which the formation of disulfide bonds is required for correct folding of some proteins.^105^, ^106^ To deal with ROS formed during protein folding in ER stress, the PERK pathway induces the transcription of glutathione *S*-transferase, NAD(P)H:quinone oxidoreductase-1, γ-glutamate cysteine ligase, and hemeoxygenase-1 to protect the cell from oxidative stress.^44^,^54^,^109^–^124^ Oxidative stress is believed to play an important role in age-related macular degeneration (AMD), retinitis pigmentosa (RP) and other ocular diseases.^125^

Some evidence suggests a role for caspase-12 as another pro-apoptotic pathway in ER stress.^126^ Caspase-12 is a member of the group I family of caspases (also known as ICE-like caspases and consists of caspase-1, 4, 5, 11, and 13). Caspase-12 is localized on the cytoplasmic side of the ER membrane^127^ and is associated with ER stress-mediated apoptosis^79^. It has been shown that activated caspase-12 translocates to the cytosol and subsequently activates caspase-9 and caspase-3.^128^ Caspase-12 activation by amyloid β (Aβ) peptide through calpain has been shown in primary neurons.^79^ In humans, the functional caspase-12 homologue is not expressed due to a mutation^129^; therefore, other caspases may play a role in apoptosis.^130^

## NEURODEGENERATIVE DISEASES AND UPR

Retinal degeneration is one of the leading causes of blindness in humans. The current hypothesis proposed for retinal degeneration elicited by ER stress is the activation of UPR pathways. Some types of retinal degenerations share similar pathological origins to neurodegenerative diseases. These include protein misfolding, aggregate formation and activation of protein degradation machinery. Furthermore, involvement of UPR has been suggested by cell culture studies and animal models.^11^,^131^ There are a few similarities found in the origins of neurodegenerative diseases and those found in retinal degenerations. A better understanding of the pathogenesis of neurodegenerative diseases could shed light on the molecular pathology of eye diseases. Neurodegenerative diseases are chronic conditions originating from mutations in genes that lead to neural cell death. In many neurodegenerative diseases, such as Alzheimer’s disease (AD), amyotrophic lateral sclerosis (ALS), Parkinson’s disease (PD), multiple sclerosis (MS) and polyglutamine diseases (which result from expansion of a polyglutamine repeat), the major molecular pathology is the formation of protein aggregates.^132^ Aggregate formation can limit or inhibit the capacity of the cell for ER-associated protein degradation (ERAD) by proteasomes. Failure of the cell to degrade misfolded aggregates induces UPR and eventually neural cell death.^133^, ^134^

Alzheimer’s disease is among the well known neurodegenerative diseases in which the PERK-EIF2α pathway is activated.^14^,^135^–^138^ Juvenile-onset Parkinson’s disease is another example of UPR-driven cell death in which mutations in Parkin, an E3 ubiquitin ligase causes ER stress.^139^,^140^ The expression of wild type Parkin is induced by ER stress to clear misfolded proteins through ERAD.^141^ The overexpression of wild type Parkin promotes survival in cells expressing mutant α-synuclein or those treated with ER stress-inducing agents.^140^–^142^ Evidence of ER stress has been reported in other forms of neurodegenerative diseases such as ALS 134, Huntington’s disease^143^–^147^ and diseases associated with Prions such as transmissible spongiform encephalopathies of Creutzfeldt-Jakob disease and Kuru^148^.

## ROLE OF UPR IN EYE DISEASES

UPR is a response to ER stress and is implemented differently depending on cell type, nature of ER stress, its magnitude and duration.^89^, ^149^, ^150^ UPR can serve to protect the cell by re-establishing ER homeostasis or it can trigger apoptosis under severe or chronic ER stress.^151^,^152^ The presence of misfolded protein is the trigger for induction of UPR. A growing number of reports suggest that misfolded proteins play a role in the pathogenesis of several eye disorders.^153^ Several studies have demonstrated the association of misfolded protein and UPR in RP. UPR might also be involved in the conversion of dry AMD to the wet form through angiogenesis stimulated by the vascular endothelial growth factor (VEGF) released from retinal pigmented epithelium (RPE) cells.^154^ UPR has also been implicated in the early onset form of Fuchs endothelial corneal dystrophy (FECD) which is the leading indication for corneal transplantation.^155^ Among other diseases that might be linked to ER stress, investigators have reported the activation of UPR by cataract-associated αA-crystallin^156^ and collagen IV^157^ mutations in animal models. Moreover, possible involvement of ER stress has been proposed in adult-onset primary open angle glaucoma (POAG).^158^ Mutations in carbonic anhydrase (CA) IV, a highly expressed enzyme in the choriocapillaris of the human eye, are associated with the RP17 form of autosomal dominant RP due to accumulation of unfolded proteins in the ER.^159^–^161^ Besides the retina, other neuronal cells are subject to induction of UPR as shown in several neurodegenerative diseases. ALS,^162^ Parkinson’s, Huntington’s and Alzheimer’s disease, and prion-related disorders are among degenerative diseases associated with UPR.^163^

The association of UPR and visual impairment has been well described in animal models of RP. Among the over 40 genes causing RP, the most common forms of autosomal dominant RP are due to mutations in rhodopsin. Mutations in rhodopsin cause the formation of misfolded proteins which induce ER stress. Enormous amounts of rhodopsin are made every day and the overload of photoreceptors with misfolded rhodopsin triggers UPR. The induction of UPR has been observed in several animal models of RP.^70^, ^164^–^166^

## UPR IN RETINAL DEGENERATION: POTENTIAL THERAPEUTIC APPROACHES

Photoreceptors synthesize a large amount of protein every day indicating the critical role for ER function in these cells. Altered expression of some molecular chaperones, the major component of protein folding and quality control in the ER, was found in proteomics of a murine model of retinal degeneration.^11^,^167^–^169^ Some mutations in rhodopsin, which result in the production of misfolded proteins, lead to retinitis pigmentosa (RP). These types of RPs have been classified as ER storage diseases.^3^ The expression level of ATF6, phosphorylated eIF2α, and CHOP in a rat model of RP expressing rhodopsin-P23H mutation were higher as compared to control animals. Overexpression of BiP in the P23H rat model led to a decrease in CHOP levels and apoptosis, and an increase in the amplitude of the electroretinogram^164^ which indicates that increased folding capacity in cells may inhibit apoptosis. The expression of ER stress markers, XBP1 and Hrd1, was increased in a fly model of RP expressing mutant Rh1, the equivalent of rhodopsin in vertebrates. It was demonstrated that reduced function of ERAD led to an increase in mutant Rh1 which suggest its role in degradation of mutant opsin. On the other hand, overexpression of the ERAD machinery reduced the levels of mutant Rh1 and markers of ER stress.^165^ These reports demonstrated induction of UPR in animal models of retinal degeneration. They also exhibited a reduction in apoptosis by overexpression of chaperones, such as BiP.

Several therapeutical approaches to alleviate retinal degeneration targeted protein folding by using chemical chaperones.^170^–^172^ Chemical chaperones are small molecules which assist protein folding by non-covalent interactions with partially folded or misfolded proteins.^64^,^172^–^176^ Protein synthesis is an energy consuming process which is assisted and monitored by several proteins mostly residing in the ER. Among assistant proteins, chaperones stabilize partially folded proteins and bind to exposed hydrophobic surfaces to direct a forming protein to its native state. Failure to adopt the native conformation will target the misfolded protein to ER-associated degradation machinery to prevent aggregate formation.^170^ However, this folding assistance and quality control might become overwhelmed due to other cellular insults such as oxidative stress, hypoxia, altered calcium homeostasis or vastly expressed inherently misfolded protein. To cope with this stress, the ER initiates a compensatory cascade of responses, i.e. the UPR,^177^ which is initially a survival response that reduces the general translation of proteins to alleviate the burden of protein folding on the ER, upregulates chaperones to facilitate protein folding and induces ER-associated degradation machinery. Failure of adopted strategies to recover from stress leads to induction of apoptosis to eliminate injured cell.^137^,^167^,^178^ Several neurodegenerative diseases are associated with misfolded proteins and consequently ER stress.^11^,^131^,^137^, ^178^

Recent studies have demonstrated facilitated protein folding by pharmacological chaperones in models of RP. Some forms of RP are associated with misfolding of mutant rhodopsin, aggregate formation and targeted degradation by the ubiquitin proteasome system. Investigators studying P23H, the most common mutation found in rhodopsin, reported that 11-cis-7-ring retinal can promote proper folding, glycosylation and localization of P23H to the cell surface.^179^–^181^ Rescue of misfolded P23H was achieved in another study by cotransfection of P23H with Hsp70, β-synuclein, or γ-synuclein chaperones in P661W photoreceptor cells and resulted in the formation of fewer inclusion bodies.^182^

Compounds known as chemical chaperones^183^,^184^ such as tauroursodeoxy-cholic acid (TUDCA) and 4-phenyl butyric acid, have origins in traditional Chinese medicine and have been used in humans for a variety of diseases including RP. Although some beneficial effects have been demonstrated, the definitive effect of chemical chaperones to inhibit ER stress-induced apoptosis has not been proven.^185^–^187^

Several investigators have shown the effectiveness of a high-throughput screen for chemical chaperones to find a potent protein aggregate inhibitor. Accordingly, yeast cells expressing Htt-103Q-EGFP (*Huntington* gene with extended polyglutamine fused to EGFP) were generated and demonstrated poor growth and low expression of the fusion protein. These cells were then treated with a library consisting of 16,000 different compounds and screened for restoration of cell growth and expression of fusion protein (Htt-103Q-EGFP). After finding effective compounds from the library, investigators examined the effect of hit compounds in the mouse and Drosophila model of Huntington’s disease.^11^ Future studies need to uncover the extent to which the UPR is induced in retinal degenerative diseases. A better understanding of the role of timing in the induction of UPR elements is crucial to target pro-apoptotic factors. High-throughput studies will play a major role in finding mechanisms of retinal degeneration and potential therapeutic compounds.
